# Nrf2 activation in the human brain after stroke due to supratentorial intracerebral haemorrhage: a case–control study

**DOI:** 10.1136/bmjno-2021-000238

**Published:** 2022-02-19

**Authors:** Edward Christopher, James J M Loan, Neshika Samarasekera, Karina McDade, Jamie Rose, Jack Barrington, Jeremy Hughes, Colin Smith, Rustam Al-Shahi Salman

**Affiliations:** 1 The University of Edinburgh College of Medicine and Veterinary Medicine, Edinburgh, UK; 2 Division of Clinical Neurosciences, NHS Lothian, Edinburgh, UK; 3 Centre for Clinical Brain Sciences, The University of Edinburgh, Edinburgh, UK; 4 Academic Neuropathology, The University of Edinburgh, Edinburgh, UK; 5 UK Dementia Research Institute, The University of Edinburgh, Edinburgh, UK; 6 Centre for Inflammation Research, The University of Edinburgh, Edinburgh, UK

**Keywords:** histopathology, stroke, neuropathology

## Abstract

**Aims:**

Pharmacological activation of the antioxidative transcription factor nuclear factor erythroid 2-related factor 2 (Nrf2) improves outcomes in experimental models of intracerebral haemorrhage (ICH). However, the Nrf2 pathway has not been previously studied in humans after ICH. Our study aims to address this gap.

**Methods:**

We selected cases with fatal ICH from a prospective community-based inception cohort study and age-matched and sex-matched controls who died suddenly of non-neurological disease. We used immunohistochemistry to quantify Nrf2 (% total area stained overall and % of nuclei stained) and CD68 expression in controls and perihaematomal, ipsilateral and contralateral brain tissue from cases. We measured downstream haem oxygenase-1 (HMOX1) and NAD(P)H dehydrogenase quinone 1 [NQO1] expression using RNA in situ hybridisation.

**Results:**

26 ICH cases (median age: 82 (IQR 76–86); 13 (50%) male) and eight controls (median age: 79 (IQR 77–80); 3 (37.5%) male) were included. We found no significant differences in overall % of Nrf2 staining between ICH cases and controls. However, the mean % of nuclei staining for Nrf2 seemed higher in perihaematomal compared with contralateral regions, although this was only statistically significant >60 days after ICH (25% (95% CI 17% to 33%) vs 14% (95% CI 11% to 17%), p=0.029). The percentage of perihaematomal tissue staining for CD68 was higher >60 days after ICH (6.75%, 95% CI 2.78% to 10.73%) compared with contralateral tissue (1.45%, 95% CI 0.93% to 1.96%, p=0.027) and controls (1.08%, 95% CI 0.20% to 1.97%, p=0.0008). RNA in situ hybridisation suggested increased abundance of HMOX1 and NQO1 transcripts in perihaematomal versus distant ipsilateral brain tissue obtained <7 days from onset of ICH.

**Conclusions:**

We found evidence of Nrf2 activation in human brain tissue after ICH. Pharmacological augmentation of Nrf2 activation after ICH might be a promising therapeutic approach.

## Introduction

Intracerebral haemorrhage (ICH) accounts for approximately 10% of strokes in high-income countries and 20% of strokes in low-income/middle-income countries.^1^ About 40% of patients die within the first month, and 86% are dead or dependent within 1 year.^2^ Acute medical or surgical treatments for ICH, supported by consistent high-quality evidence from randomised controlled trials, remain to be found.^3^ In ageing populations, the incidence of ICH is projected to rise,^4^ making the identification of therapeutic strategies for ICH important.

The pathophysiology of brain injury due to ICH includes haematoma expansion, mass effect, altered cerebral haemodynamics, thrombin and iron toxicity, and inflammation via multiple pathways, with one common pathway being oxidative stress.^5^ Nuclear factor erythroid 2-related factor 2 (Nrf2) is a basic leucine zipper transcription factor, which regulates antioxidant response element (ARE) gene expression^6^ and is selectively expressed in non-neuronal cells.^6 7^ Mononuclear phagocytes (MNPs)—microglia and macrophages—are the major cell types to express Nrf2 in the human brain.^8^ Under quiescent conditions, Nrf2 is maintained in the cytoplasm by the ubiquitin ligase Kelch-like ECH-associated protein 1 (KEAP1).^9^ Oxidative stress causes dissociation of Nrf2 from the Nrf2-KEAP1 complex.^6^ Nrf2 is then transported to the nucleus, where it modulates transcription of various genes including NAD(P)H dehydrogenase quinone 1 (NQO1) and haem oxygenase-1 (HMOX1) to induce an overall cytoprotective transcriptional profile^9 10^ (online supplemental figure 1).

10.1136/bmjno-2021-000238.supp1 Supplementary data

Nrf2 activates AREs in MNPs and facilitates erythrocyte phagocytosis and clearance.^11^ Nrf2 activation improves outcomes in animal models of ICH by increasing rates of haematoma clearance and enhancing antioxidant defences; Nrf2 agonists have also consistently improved outcomes in these animal models through similar mechanisms.^11–15^ Nrf2 agonists may, therefore, have therapeutic potential for improving recovery after ICH in humans. However, there are no published studies directly examining the Nrf2 pathway in human brain tissue after ICH.^16^ This is important to establish the translational relevance of preclinical experimental studies of Nrf2 activation after ICH.

Therefore, we aimed to quantify the activity of the Nrf2 pathway in brain tissue and nuclei after ICH in humans and explore its activity over time after ICH onset. Because Nrf2 is most highly expressed in MNPs in the human brain, we also sought to describe perihaematomal changes in expression of the MNP marker CD68 following ICH.^17 18^

## Methods

### Study design

In this case–control study, cases were patients with first-ever spontaneous supratentorial ICH between 2010 and 2016 ascertained by a prospective, community-based, inception cohort study of adults (aged ≥16 years old) in the Lothian health board region of Scotland,^19^ with authorisation for research autopsy as part of the Lothian Intracerebral Haemorrhage, Pathology, Imaging and Neurological outcome study. Controls were people who died suddenly in 2005–2014 of non-neurological causes, who underwent autopsy to determine the cause of death, and authorisation had been provided for research autopsy for the Sudden Death Brain and Tissue Bank. Both brain banks include adults from the Lothian health board region of Scotland and are part of the Medical Research Council Edinburgh Brain and Tissue Bank.^19 20^

### Setting

Academic tertiary medical centre situated in Edinburgh and all other secondary and primary care centres around the health board region of Lothian, Scotland. The study described in this manuscript was conducted between 2016 and 2021.

### Participants

Blinded to histological findings, EC and CS selected 26 cases with first-in-a-lifetime spontaneous ICH (not intracranial haemorrhage that was exclusively extra-axial or due to trauma, macrovascular causes, neoplasms or haemorrhagic transformation of an ischaemic stroke): 8 died <7 days after ICH onset, 9 died 7–60 days after ICH onset and 9 died >60 days after ICH onset. Cases were selected such that the similarity of the age, sex and ICH epicentre distribution (ie, lobar vs deep, as defined previously^19^) of the three time groups was preserved as much as possible. Blinded to histological findings, EC and CS selected eight controls so that they had a similar age and sex distribution to cases, without a clinical history or macroscopic pathological evidence of ICH, from the Sudden Death Brain and Tissue Bank. Written consent was provided by participants, or their legal representative if participants lacked mental capacity, in accordance with the Human Tissue (Scotland)Act 2006 and the Adults with Incapacity (Scotland) Act 2000. Written consent was provided by the participant’s nearest relative in all but one case; in this case, written consent was obtained from the patient themselves prior to their death. Further details about the consenting process were described in previous studies.^19 20^ Briefly, eligible patients and/or their families were identified and approached if appropriate by a doctor or highly trained research nurses about the possibility of tissue donation for research purposes. Information about the projects, their aims and the process of tissue donation was provided with full opportunities for questions.

### Variables

We collected demographic details and potential confounders (such as neurological comorbidities and medication use at the time of ICH for cases (or time of death for controls), which might affect Nrf2 activation^21 22^) from patients’ medical records or their relatives. The assessment of brain tissue histology was blinded to these clinical variables. Our outcomes of interest are further described in the subsequent sections of the manuscript.

### Data sources/measurement

For each case, a consultant neuropathologist (CS) identified brain areas to sample from formalin-fixed, paraffin-embedded tissue as ‘perihaematomal’ (adjacent to the ICH), ‘ipsilateral’ (distant from the ICH, but in the same cerebral hemisphere) and ‘contralateral’ (in the opposite cerebral hemisphere to the ICH) (online supplemental table 1). The4 µm sections of tissue were immunohistochemically stained for Nrf2 (rabbit polyclonal anti-Nrf2 antibodies, 1:50 (ab31163, Abcam, UK)) and CD68 to detect phagocytic activity in reactive myeloid cells (microglia and macrophages) (mouse monoclonal anti-CD68 antibodies, 1:100, citric acid antigen retrieval (M0876, Dako, Denmark)). We stained positive and negative controls for Nrf2 and CD68 antibodies to enable concurrent comparison when visually inspecting slides. The Novolink Max Polymer Detection System (RE7280-K, Leica, Germany) was used in accordance with the manufacturers’ instructions. Slides were developed with 3,3’-diaminobenzidine (DAB) and weakly counterstained with haematoxylin to facilitate higher sensitivity of image analysis. Image analysis was undertaken with an automated slide scanner (Axio Scan Z1, Zeiss, Germany) linked to the Zen software (Zeiss, Germany). Thirty-two randomly placed coloured images from each slide from ICH cases and controls were captured at ×40 magnification. Image capture settings and calibration parameters remained unchanged throughout and no manipulation was performed on the images. EC measured the % of each image area that stained positively with each antibody, using the Trainable Weka Segmentation feature of ImageJ (V.1.8.0_66; https://imagej.nih.gov/ij/), which achieves complete binary segmentation of images into areas stained or not stained with antibody. EC determined the overall % of each image area that are stained for Nrf2 +or CD68+using the ‘analyse particles’ feature of ImageJ without restrictions on particle size or circularity limit. EC was trained to identify positive and negative DAB nuclear staining by a consultant neuropathologist (CS). EC subsequently quantified Nrf2 nuclear localisation by counting the number of nuclei that were either positive or negative for Nrf2 staining using the Cell Counter feature on ImageJ. The percentage of nuclei that was positive was calculated by dividing the number of positive nuclei by the total number of nuclei in each image multiplied by 100 and the mean was calculated from the randomly placed images for each case and control. EC was blinded to the details of the image (eg, whether it was a case or a control, whether it was from a perihaematomal, ipsilateral or contralateral region) throughout and quantification was performed in a random order using online random sequence generator.

For in situ RNA hybridisation to assess HMOX1 and NQO1, which are canonical Nrf2-regulated genes through ARE binding motifs,^23^ two cases who died <7 days after ICH, two cases who died between 7 and 60 days after ICH, and one case who died later than 60 days after ICH were randomly selected from the existing cases. Two controls were also randomly selected from the existing controls. Sections of perihaematomal and distant ipsilateral tissue were selected from ICH cases. The 4 µm thick sections of formalin-fixed paraffin-embedded tissues were stained with fast red targeted either to HMOX1 or NQO1 mRNA using BaseScope probes (ACDBio) in one batch. Tissue was deparaffinised and treated with proteinase 3 before the probe solution was applied. Eight rounds of amplification at 40°C were performed prior to chromogen application. All sections were lightly counterstained with haematoxylin. Ten non-overlapping x40 fields of view were acquired per slide using a Zeiss AxioImager 2 upright microscope. Fast red staining was quantified on Fiji (V.2.0. 2018) using the default FastRed colour deconvolution vector, followed by serial minimal thresholding and Gaussian blur subtraction (σ=60). Smoothing of the final image was achieved with a further Gaussian blur (σ=2), residual background subtracted with a rolling ball method. The image was autothresholded using ‘Moments’ and then watershed. Fast red positive dots and clusters were quantified using ‘analyse particles’ with circularity=0.1–1.

### Bias

We blinded the selection of cases and controls to histological findings and blinded the collection of histological data to clinical variables. Throughout the study, cases and controls were deidentified and coded such that assessments were blind to their identity. The cases’ and controls’ clinical variables and histological data were kept in separate spreadsheets and were only linked for data analysis purposes. Cases and controls for in-situ RNA hybridisation were randomly selected.

### Study size

Given the absence of published studies on Nrf2 pathway activity after ICH in human ICH cases or sudden death controls and activity of the Nrf2 pathway over time after ICH, we did not have prior estimates on which to base a sample size calculation. Therefore, we included all controls available in our brain bank with age and sex distributions similar to those of patients incident with first-ever ICH in our population,^24^ and used a case–control ratio of approximately 3:1 to maximise power. Human brain tissue is a scarce resource, and our case–control ratio is the best our tissue bank allows without compromising comparability of cases and controls.

### Statistical methods

EC and JL used GraphPad Prism (V.8.4.1) to summarise distributions of variables (mean per patient, with error bars representing the 95% CI of the mean) and perform statistical tests. Differences in distributions between the means of controls and cases were compared using the Kruskal-Wallis test and corrected Dunn’s post hoc test, which corrects for multiple comparisons. All ICH time point groups (ie, died <7 days, 7–60 days and >60 after ICH onset) were included in each analysis. Differences in distributions between the means of each group of cases (according to time from ICH onset and brain location—using contralateral tissue as the comparator) were tested for statistical significance by using matched two-way analysis of variance with the Geisser-Greenhouse correction and the Dunnet test, which corrects for multiple comparisons. All statistical tests were two tailed. Differences were considered statistically significant at p<0.05.

## Results

### Clinical characteristics of cases and controls

We selected 26 ICH cases (median age 82 (IQR 76–86); 13 (50%) male; 14 deep ICH epicentre and 12 lobar ICH epicentre; online supplemental table 2) and 8 sudden death controls (median age 79 (IQR 77–80) years; 3 (37.5%) male; online supplemental table 3). One of the cases had hepatocellular cancer diagnosed 1 month before ICH, and two controls died from complications of metastatic cancers. One of the controls was taking prednisolone at the time of death but none of the other controls or cases took immunosuppressive medication. Ten (38.5%) cases, but none of the controls, had dementia at the time of death.

### Nrf2 tissue and nuclear staining

**Figure 1:**
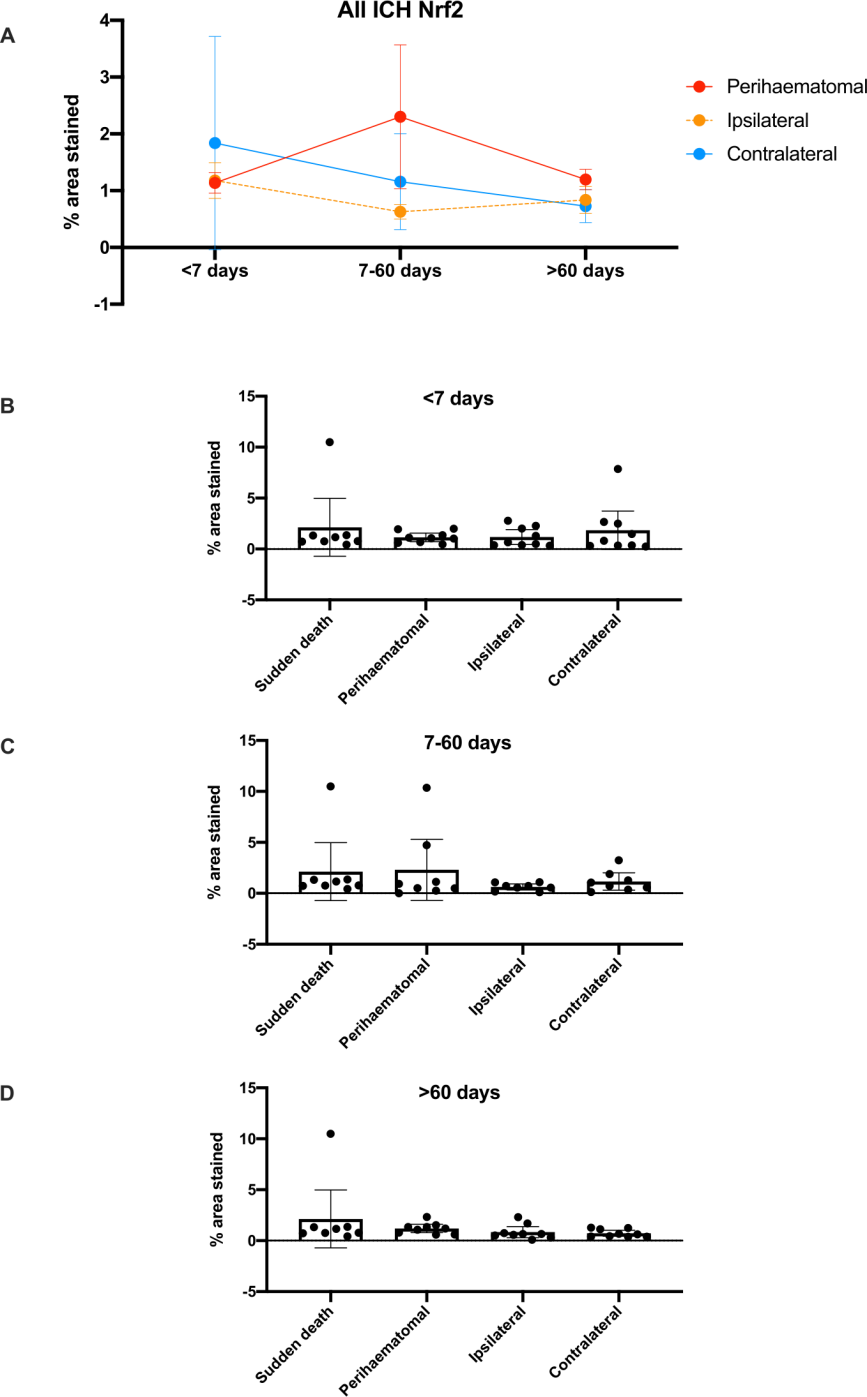
Mean (95% CI) % area stained positive for Nrf2 in ICH tissue by time of death after ICH symptom onset (A) and by location in relation to the ICH epicentre as well as time of death after ICH symptom onset, compared with sudden death controls (B–D). ICH, intracerebral haemorrhage; Nrf2, nuclear factor erythroid 2-related factor 2.

**Figure 2:**
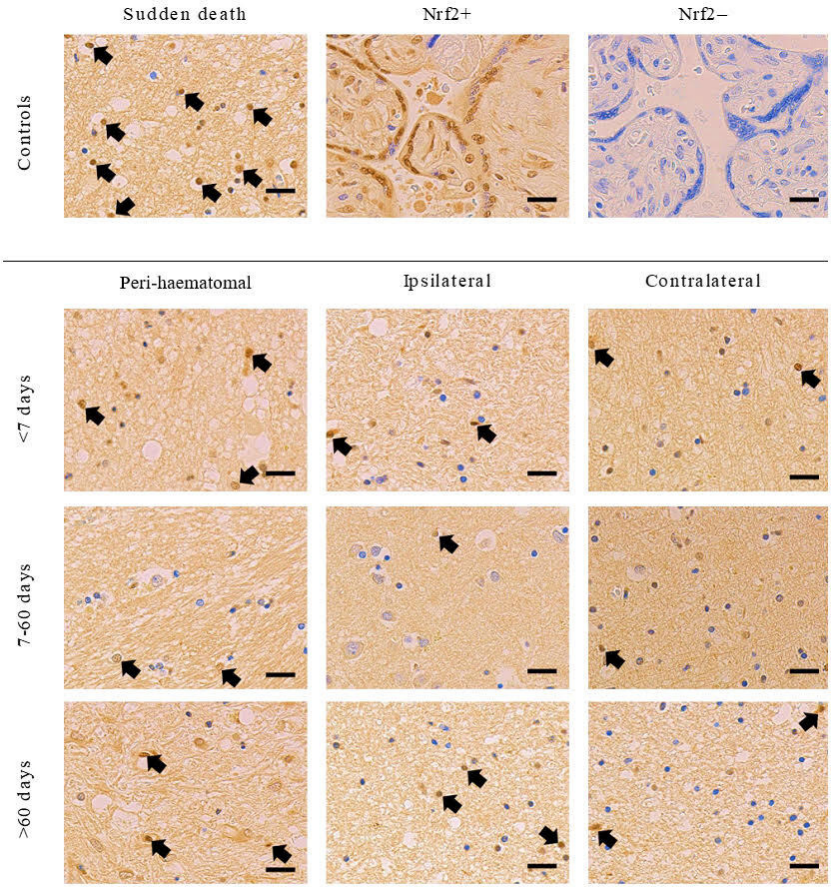
Representative images of Nrf2 immunohistochemical staining from ICH cases and sudden death controls included in the study. Nrf2 + and Nrf2– controls (human placenta) are shown on top row of middle and right column, respectively, as positive and negative controls. Images are all without manipulation and under the same magnification. Contrast was equally enhanced in all images to aid visualisation. Black arrows exemplify Nrf2 +nuclei; not all Nrf2 +nuclei were highlighted in the interest of clarity. Scale bars=20 µm. ICH, intracerebral haemorrhage; Nrf2, nuclear factor erythroid 2-related factor 2.

**Figure 3:**
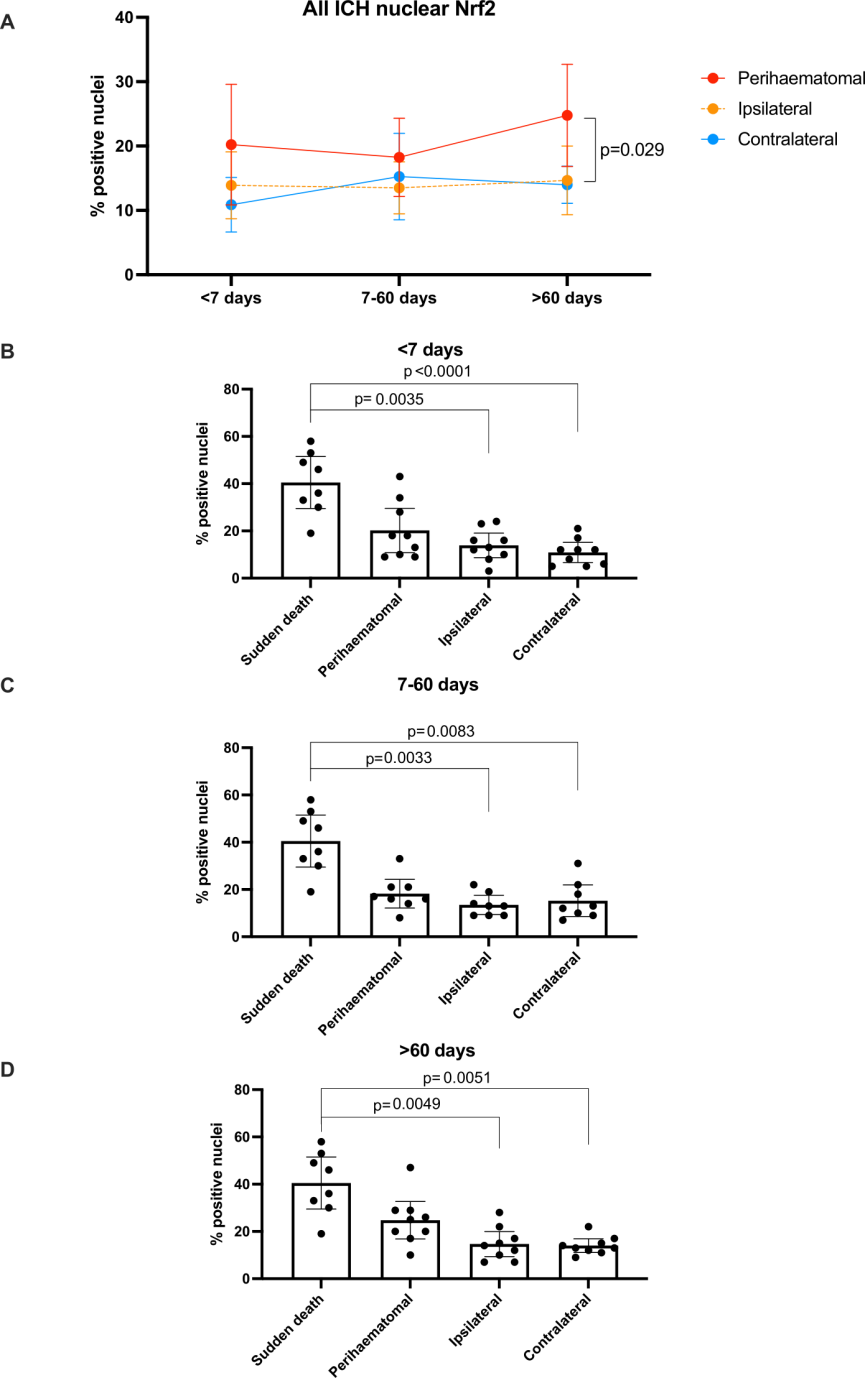
Mean (95% CI) % of nuclei stained positive for Nrf2 in ICH tissue by time of death after ICH symptom onset (A) and by location in relation to the ICH epicentre as well as time of death after ICH symptom onset, compared with sudden death controls (B–D). ICH, intracerebral haemorrhage; Nrf2, nuclear factor erythroid 2-related factor 2.

There were no differences in the percentage total area staining positive for Nrf2 overall in brain tissue sections between cases and controls, nor between brain areas in ICH cases, nor between ICH cases according to their time of death after ICH onset (table 1; figure 1). When quantifying only nuclear staining for Nrf2 (figure 2), we found that the mean % of nuclei staining for Nrf2 seemed higher in peri-haematomal regions compared with contralateral regions, although this was only statistically significant >60 days after ICH onset (25% (95% CI 17% to 33%) vs 14% (95% CI 11% to 17%), p=0.029; table 1). The percentage of nuclei staining for Nrf2 was lower in ICH cases in ipsilateral and contralateral brain regions at all times after ICH onset compared with sudden death controls (figure 3).

**Table 1:** Mean measures (95% CI) of % area staining for Nrf2, % of nuclei staining for Nrf2 and % area staining for CD68 in supratentorial ICH cases and sudden death controls

ICH cases									Sudden death controls
Time after onset (days)	Perihaematomal tissue	Ipsilateral tissue	Contralateral tissue	Perihaematomal versus contralateral p value	Ipsilateral versus contralateral p value	Sudden death versus perihaematomal p value	Sudden death versus ipsilateral p value	Sudden death versus contralateral p value	
% area staining for Nrf2									
<7	1.14 (0.72 to 1.56)	1.18 (0.45 to 1.91)	1.84 (–0.04 to 3.72)	0.61	0.58	0.99	0.99	0.99	2.13 (–0.71 to 4.97)
7–60	2.30 (−0.70 to 5.30)	0.62 (0.32 to 0.93)	1.16 (0.31 to 2.01)	0.41	0.29	0.99	0.69	0.99	
>60	1.20 (0.78 to 1.61)	0.84 (0.29 to 1.38)	0.73 (0.44 to 1.02)	0.15	0.86	0.99	0.99	0.99	
% nuclei staining for Nrf2									
<7	20 (11 to 30)	14 (9 to 19)	11 (7 to 15)	0.056	0.13	0.086	0.0035	<0.0001	41 (29 to 52)
7–60	18 (12 to 24)	14 (5 to 17)	15 (9 to 22)	0.60	0.79	0.16	0.0033	0.0083	
>60	25 (17 to 33)	15 (9 to 20)	14 (11 to 17)	0.029	0.95	0.99	0.0049	0.0051	
% area staining for CD68									
<7	1.47 (0.94 to 2.00)	1.38 (0.97 to 1.78)	1.35 (0.98 to 1.78)	0.96	0.99	0.99	0.99	0.99	1.08 (0.20 to 1.97)
7–60	2.53 (1.14 to 3.92)	1.04 (0.45 to 1.63)	1.22 (0.73 to 1.72)	0.14	0.56	0.13	0.99	0.99	
>60	6.75 (2.78 to 10.73)	0.94 (0.56 to 1.32)	1.45 (0.93 to 1.96)	0.027	0.16	0.0008	0.99	0.99	

Reported p values reflect comparisons of tissue adjacent (peri-haematomal) or distant but in the same cerebral hemisphere (ipsilateral) to ICH versus tissue in the cerebral hemisphere unaffected by ICH (contralateral).

ICH, intracerebral haemorrhage; Nrf2, nuclear factor erythroid 2-related factor 2.

### RNA in situ hybridisation

**Figure 4:**
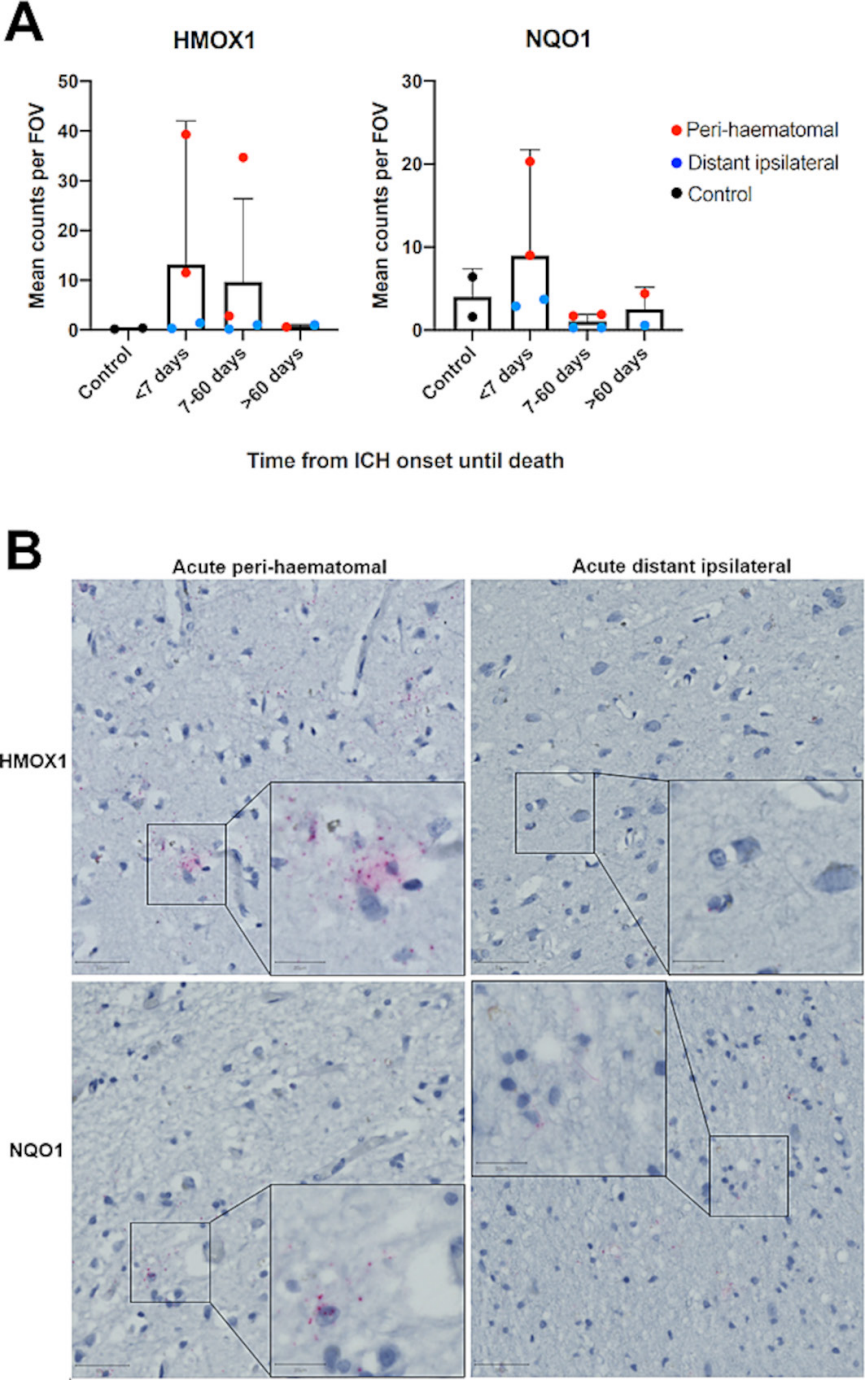
(A) Mean (95% CI) in situ RNA hybridisation transcript counts per field of view (FOV) in sudden death controls versus ICH cases, by time of death after ICH symptom onset. Red points indicate perihaematomal and blue distant ipsilateral. Error bars: 95% CI. (B) Representative images of tissue from acute (<7 days from ICH onset until death) perihaematomal and distant ipsilateral tissue stained using fast red following RNA in situ hybridisation for HMOX1 or NQO1. Pink dots indicate transcripts haematoxylin counterstain. Scale bars=50 µm (main) and 20 µm (inset). HMOX1, haemoxygenase-1; ICH, intracerebral haemorrhage; NQO1, NAD(P)H dehydrogenase quinone 1.

Consistent with increased perihaematomal Nrf2 nuclear translocation, we found evidence of target gene induction in HMOX1 and NQO1 downstream of Nrf2; staining of both transcripts was higher in perihaematomal versus distant ipsilateral brain tissue obtained <7 days from onset of ICH (figure 4).

### CD68 tissue staining

**Figure 5:**
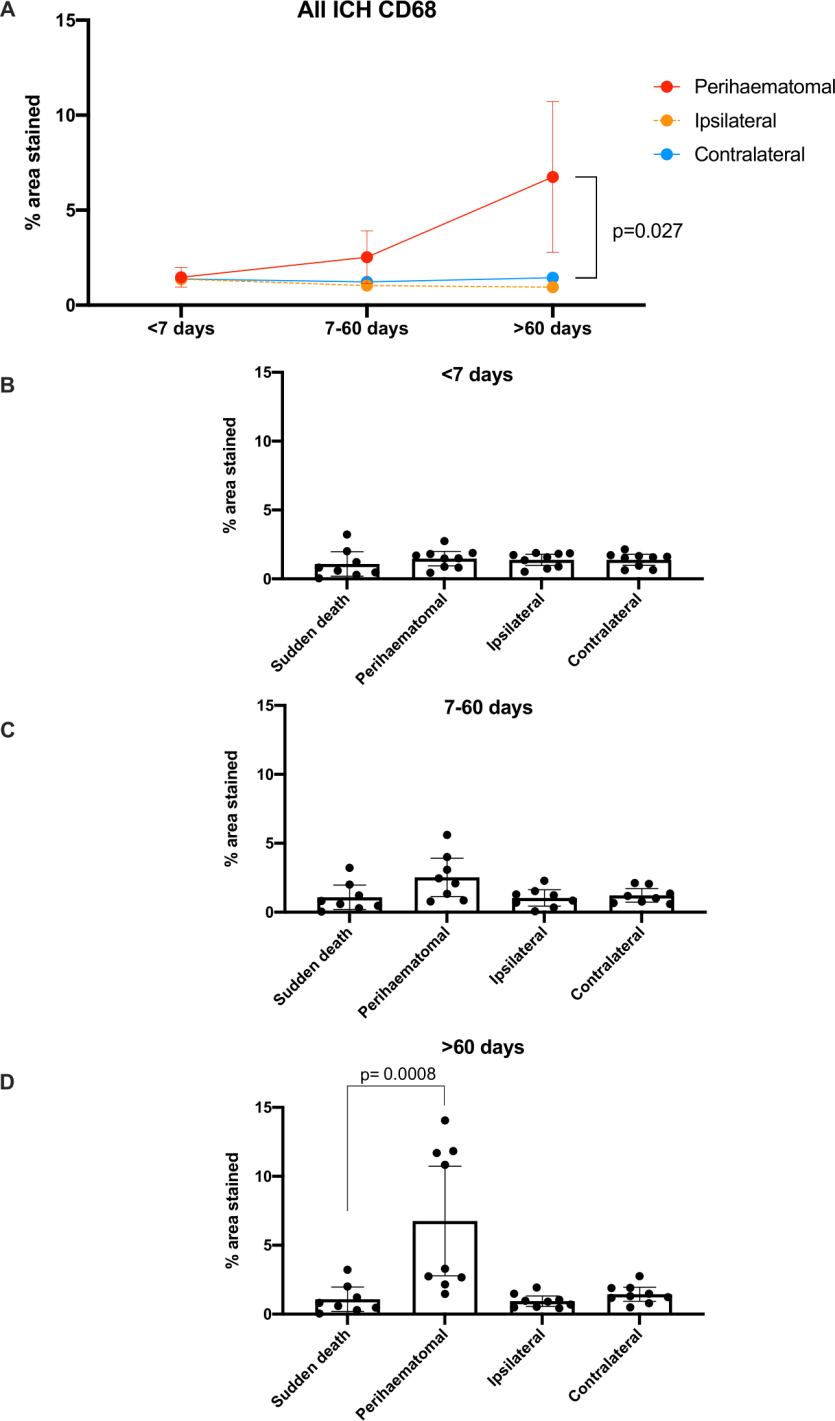
Mean (95% CI) % area stained positive for CD68 in ICH tissue by time of death after ICH symptom onset (A) and by location in relation to the ICH epicentre as well as time of death after ICH symptom onset, compared with sudden death controls (B–D). ICH, intracerebral haemorrhage.

We found differences in the % total area staining positive for CD68 overall in perihaematomal tissue >60 days after ICH (6.75% (95% CI 2.78% to 10.73%)) compared with contralateral brain tissue (1.45% (95% CI 0.93% to 1.96%), p=0.027) and compared with brain tissue from sudden death controls (1.08% (95% CI 0.20% to 1.97%), p=0.0008; table 1, figure 5 and online supplemental figure 2).

## Discussion

To our knowledge, this is the first community-based case–control study of the Nrf2 pathway after stroke due to supratentorial ICH in humans, compared with controls who died suddenly of other causes. We did not find significant differences in the percentage total area staining positive for Nrf2 overall in brain tissue sections between cases and controls. The percentage of nuclei staining for Nrf2 was lower in ICH cases in ipsilateral and contralateral brain regions at all times after ICH onset compared with sudden death controls. In ICH cases, the mean percentage of nuclei staining for Nrf2 seemed higher in peri-haematomal regions compared with contralateral regions, although this was only statistically significant >60 days after ICH onset; expression of Nrf2 target genes seemed to reflect these findings. Alongside these novel findings, the percentage total area staining positive for CD68 overall in perihaematomal tissue was higher >60 days after ICH compared with contralateral brain tissue and compared with brain tissue from sudden death controls, reflecting changes to the composition and phenotype of MNPs which may be attributable to increased phagocytic activity that could be expected in a chronic response to ICH.

Nrf2 has a role in myelomononuclear phagocytosis and haematoma clearance after experimental ICH.^11^ There is experimental and observational clinical evidence that ICH clearance reduces neuronal damage by removing toxic chemicals and relieving local ischaemia.^25–27^ This stresses the need to explore endogenous mechanisms to clear ICH and mitigate toxicity, potentially via the Nrf2 pathway. In addition to enhancing phagocytic functions of microglia, Nrf2 activation may optimise secretory profiles of monocytes to reduce brain inflammation and improve outcome in animal models.^28^ In addition to microglia, Nrf2 also influences various other cell types including astrocytes and the neurovascular unit to maintain cerebral blood flow and improve cell survival following injury.^6^ The pleiotropic properties of Nrf2, coupled with its anti-inflammatory and antioxidative effects and the availability of clinically licensed Nrf2 agonists with tolerable safety profiles, makes the Nrf2 pathway an attractive therapeutic target for improving recovery after ICH in humans.

We found evidence of Nrf2 activation in human brain tissue after ICH, although less than controls who died suddenly of other causes. This suggests, however, that the nuclear translocation of Nrf2 after ICH is submaximal, and therapeutic augmentation of this might be a promising therapeutic strategy. Some Nrf2 agonists are already clinically licensed for use in other conditions (eg, dimethyl fumarate for multiple sclerosis^29^). These therapeutics could be repurposed for clinical trials of ICH, thus expediting bench-to-bedside translation.

This study has several strengths. We minimised selection bias by identifying ICH cases in an all-inclusive, prospective, community-based, inception cohort study, with brain tissue available in a unique nested brain bank.^30^ We identified ICH-free controls from the same population, who died suddenly and had brain tissue acquired and processed using a standardised protocol.^20^ We selected cases and controls blinded to histological findings, minimised recall bias by using clinical records to collect clinical variables, and maximised statistical power by including all available controls with a ratio of cases to controls of approximately 3:1.

### Limitations

Time intervals from ICH onset to death were quite broad and future studies with narrower time intervals might generate a more precise temporal profile of Nrf2 pathway and MNP activities after ICH. Our finding of increased perihaematomal Nrf2 nuclear localisation in patients which died >60 days after ICH onset suggests that Nrf2 continues to play a role well into the chronic recovery phases of ICH. However, more than half of the group of patients which died >60 days after ICH onset died more than 6 months following their ICH. Exploratory analysis of this subgroup showed a slight trend towards a decline in nuclear localisation of Nrf2 with increasing time. This suggestion towards reduced Nrf2 nuclear localisation after 6 months from ICH onset needs to be confirmed in dedicated, adequately powered, analyses of long-term Nrf2 response to ICH.

We were unable to determine whether Nrf2 activity was less after ICH compared with control tissue because of patients’ age or other modifiers of response to ICH, or whether Nrf2 activation in controls was due to an immediate response to sudden death. High levels of nuclear translocation of Nrf2 after sudden death may reflect the oxidative stress generated by acute global hypoxia.^31^ In electrophilic environments, dissociation of Nrf2 from KEAP1 is a passive process and may occur postmortem, contrasting with the energy-demanding processes of DNA transcription to produce mRNA.^32 33^ Thus, sudden death may result in nuclear translocation of Nrf2 with a failure to affect a transcriptional response. Furthermore, evaluating the ‘normal’ resting expression and activation of Nrf2 and CD68 in human brains is challenging; data from animal models and human studies suggest that expression of both is linked to ageing (Nrf2 activity declines whereas CD68 expression increases with age^34–36^) and is associated with common neurodegenerative disorders such as Alzheimer’s disease and Parkinson’s disease.^37 38^ Even fresh sections of ‘normal’ brain tissue obtained during neurosurgery are subject to oxidative and inflammatory stress, and this is particularly challenging when evaluating metabolic and inflammatory sensors such as Nrf2.^39^ We attempted to minimise confounding factors by matching our cases for age, sex, and choosing equal numbers of deep and lobar ICH locations. Although baseline characteristics were similar between cases and controls, there was an imbalance in dementia which was unavoidable due to the limited availability of tissue and the frequency of cerebral small vessel disease as an underlying cause of both ICH and vascular dementia. Since nearly 40% of our cases had dementia, this might have increased the expression of Nrf2 target genes in cases,^37^ although we did not find evidence of this in an exploratory analysis of Nrf2 nuclear localisation stratified by severity of cerebral amyloid angiopathy (online supplemental figure 3). Future studies should try to match cases and controls for dementia or burden of cerebral small vessel disease if possible.

The meaning of the apparently ‘delayed’ Nrf2 nuclear staining following ICH observed in our study is unclear. A type 1 error due to imprecision is the most likely explanation for the lack of significance at earlier time points, given that the point estimates of the mean in perihaematomal tissue are greater than in other regions at all other time points (figure 3A). In addition, the 95% CI around the means of the perihaematomal regions are notably wider than ipsilateral or contralateral regions; there was greater variability in perihaematomal regions compared with other regions, which may be another reason for the lack of significance. Further work with larger samples is needed to identify which subgroups have greater Nrf2 nuclear localisation.

Finally, although most deaths (73.1%) in our ICH cases were directly attributed to the ICH, the remaining deaths (26.9%) were directly attributed to pneumonia (online supplemental table 2). Research into the effects of systemic infection or metabolic derangements on Nrf2 activity in the human brain is currently extremely limited and their influence on our findings thus remains uncertain.

## Conclusion

In conclusion, we found evidence of Nrf2 activation in human brain tissue after ICH. This was less than that of controls who died suddenly of other causes and the reason for this remains to be determined. Pharmacological augmentation of the Nrf2 pathway, which is submaximally active after ICH, might therefore be a useful therapeutic strategy. We plan on undertaking deeper analysis of the transcriptional consequences of Nrf2 activation in the context of ICH using further in situ RNA hybridisation or transcriptome-wide analyses and assess the functional impact of Nrf2 activation using preclinical models of Nrf2 inhibition or activation in specific cell types in order to further dissect the findings from this study.

## Data Availability

Data are available on reasonable request. The data that support the findings of this study are available on reasonable request from the corresponding author. The data are not publicly available due to privacy or ethical restrictions.
