# Supplementary material for: Nrf2 activation in the human brain after stroke due to supratentorial intracerebral haemorrhage: a case–control study
Source: BMJ Neurol Open. 2022 Feb 19;4(1):e000238. doi: 10.1136/bmjno-2021-000238 (PMC8860052; doi:10.1136/bmjno-2021-000238)
Supplement: Supplementary data [file bmjno-2021-000238supp001.pdf]

	Reference Numbers	Peri-haematomal	Ipsilateral	Contralateral
ICH epicentre recorded as **lobar (frontal)**	044/14	BA24 Anterior cingulate gyrus (R)	BA17 Occipital (R)	BA17 Occipital (L)
	043/14	BA46 Frontal convexity (R)	BA19 Occipital (R)	BA19 Occipital (L)
	012/12	BA24 Anterior cingulate gyrus (R)	Periventricular white matter (R)	Periventricular white matter (L)
	007/13	BA24 Anterior cingulate gyrus (L)	Amygdala (L)	Amygdala (R)
	038/10	Anterior hippocampus (L)	BA24 Anterior cingulate gyrus (L)	BA24 Anterior cingulate gyrus (R)
	027/10	BA8 Anterior frontal convexity (R)	Posterior hippocampus (R)	Posterior hippocampus (L)
	034/11	BA8 Anterior frontal convexity (R)	Anterior hippocampus (R)	Anterior hippocampus (L)
	017/14	BA4 Frontal motor (R)	BA19 Occipital (R)	BA19 Occipital (L)
	040/14	BA46 Frontal convexity (R)	BA19 Occipital (R)	BA19 Occipital (L)
	034/12	BA11 Inferior frontal (L)	Amygdala (L)	Amygdala (R)
	051/13	BA8 Anterior frontal convexity (R)	BA19 Occipital (R)	BA19 Occipital (L)
	010/13	BA11 Inferior frontal (L)	Amygdala (L)	Amygdala (R)
ICH epicentre recorded as **deep (basal ganglia)**	022/15	Basal ganglia mammillary body (L)	BA19 Occipital (L)	BA19 Occipital (R)
	034/16	Basal ganglia mammillary body (L)	BA19 Occipital (L)	BA19 Occipital (R)
	013/13	Basal ganglia mammillary body (R)	Posterior hippocampus (R)	Posterior hippocampus (L)
	019/12	BA41/42 Superior temporal gyrus (R)*	Periventricular white matter (R)*	Hypothalamus (L)
	029/12	BA44/45 Broca's area (L)*	Amygdala (L)*	Amygdala (R)
	060/14	Basal ganglia mammillary body (L)	BA19 Occipital (L)	BA19 Occipital (R)
	037/13	BA20/21 Inferior temporal gyrus (L)*	Occipital white matter (L)*	Occipital white matter (R)
	014/12	Frontal white matter (R)*	Periventricular white matter (R)*	Periventricular white matter (L)
	038/14	Basal ganglia mammillary body (L)	BA19 Occipital (L)	BA19 Occipital (R)
	024/16	Basal ganglia mammillary body (L)	BA19 Occipital (L)	BA19 Occipital (R)
	054/12	BA38 Temporal tip (L)	Amygdala (L)	Amygdala (R)
	001/13	Anterior hippocampus (R)	Amygdala (R)	Amygdala (L)
	015/11	Anterior hippocampus (L)	Amygdala (L)	Amygdala (R)
	006/12	BA20/21 Interior temporal gyrus (L)*	Periventricular white matter (L)*	Periventricular white matter (R)
**Controls** (sudden death)	042/14		BA9 Anterior frontal parasagittal (R)*	
	014/13		BA9 Anterior frontal parasagittal (L)*	
	027/11		BA9 Anterior frontal parasagittal (L)	
	013/09		BA11 Inferior frontal (L)	
	039/05		BA9 Anterior frontal parasagittal (L)	
	050/05		BA9 Anterior frontal parasagittal (R)	
	009/09		BA46 Frontal convexity (R)	
	002/06		BA46 Frontal convexity (L)	

Supplementary table 1. Brain regions of ICH cases and sudden death controls included in the study. Area selections and reference numbers were unblinded for this table but otherwise blinded throughout the study. BA=Brodmann area, L=left; R=right. *Tissue selected for RNA in-situ hybridisation analysis.

Case	Age at death (years)	Sex (M/F)	Dementia before ICH	ICH to death (days)	Post-mortem interval (hours)	ICH site	Cerebral amyloid angiopathy rating of lobar brain areas used*	Disease or condition leading directly to death**
Died within 7 days of ICH onset								
044/14	91-95	F	Dementia	0	118	Lobar	Absent	ICH
043/14	76-80	F	No dementia	3	40	Lobar	Mild	ICH
012/12	86-90	F	No dementia	1	155	Lobar	Mild	ICH
022/15	61-65	M	Dementia	3	28	Deep		Aspiration pneumonia
034/16	76-80	F	No dementia	2	68	Deep		ICH
013/13	86-90	M	No dementia	3	53	Deep		ICH
019/12	81-85	M	No dementia	3	107	Deep		ICH
029/12	41-45	M	No dementia	2	98	Deep		ICH
Died between 7-60 days from ICH onset								
007/13	81-85	M	Dementia	8	88	Lobar	Moderate-Severe	ICH
038/10	66-70	F	Dementia	9	66	Lobar	Severe	ICH
027/10	86-90	M	No dementia	21	93	Lobar	Moderate-Severe	ICH
034/11	81-85	F	No dementia	44	40	Lobar	Severe	ICH
060/14	91-95	F	No dementia	8	41	Deep		ICH
037/13	71-75	M	No dementia	10	52	Deep		ICH
014/12	76-80	M	Dementia	21	36	Deep		Bronchopneumonia
038/14	86-90	F	Dementia	41	59	Deep		ICH
024/16	86-90	F	Dementia	58	54	Deep		Hospital-acquired pneumonia
Died later than 60 days from ICH onset								
017/14	71-75	M	Dementia	110	58	Lobar	Severe	ICH
040/14	86-90	F	No dementia	139	90	Lobar	Severe	ICH
034/12	76-80	M	No dementia	270	107	Lobar	Severe	ICH
051/13	96-100	F	No dementia	932	36	Lobar	Mild	Bronchopneumonia
010/13	81-85	M	No dementia	996	84	Lobar	Severe	ICH
054/12	66-70	M	No dementia	64	188	Deep		Bronchopneumonia
001/13	71-75	M	Dementia	161	95	Deep		ICH
015/11	76-80	F	No dementia	265	49	Deep		Bronchopneumonia
006/12	91-95	F	Dementia	492	73	Deep		Bronchopneumonia

Supplementary table 2. Demographic characteristics of the ICH cases included in the study. M=male, F=female. Age at death is provided as a range to maximise anonymity.

**Cerebral amyloid angiopathy rating was as previously defined in Rodrigues et al. The Edinburgh CT and genetic diagnostic criteria for lobar intracerebral haemorrhage associated with cerebral amyloid angiopathy: model development and diagnostic test accuracy study. The Lancet Neurology. March 2018; 17(3): 232-240.*

***This information was ascertained from patients' Medical Certificates of Cause of Death (MCCDs) and was determined by the senior clinician responsible for the patient's care supported by independent review of patients' clinical records.*

Control	*Age at death (years)*	*Sex (M/F)*	*Cause of death*	*Post-mortem interval (hours)*
042/14	61-65	M	metastatic renal cell carcinoma	76
014/13	71-75	F	pulmonary thromboembolism	41
027/11	76-80	F	ruptured aneurysm of aortic root	46
013/09	76-80	F	ischaemic heart disease	45
039/05	76-80	F	metastatic colon carcinoma	100
050/05	81-85	M	aortic dissection	44
009/09	81-85	F	myocardial infarction	50
002/06	76-80	M	bronchopneumonia	95

Supplementary table 3. Demographic characteristics of the sudden death controls included in the study. M=male, F=female. Age at death is provided as a range to maximise anonymity.

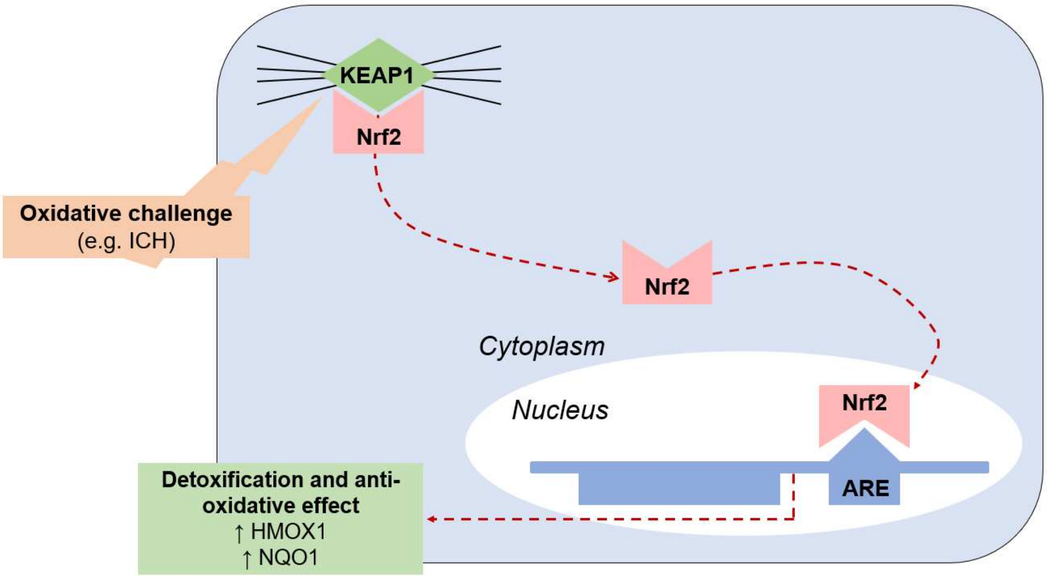

Supplementary figure 1. Simplified diagram illustrating the role of Nrf2 as master regulator of endogenous antioxidant defence pathway. The dashed line illustrates the fate of Nrf2 under oxidative conditions.

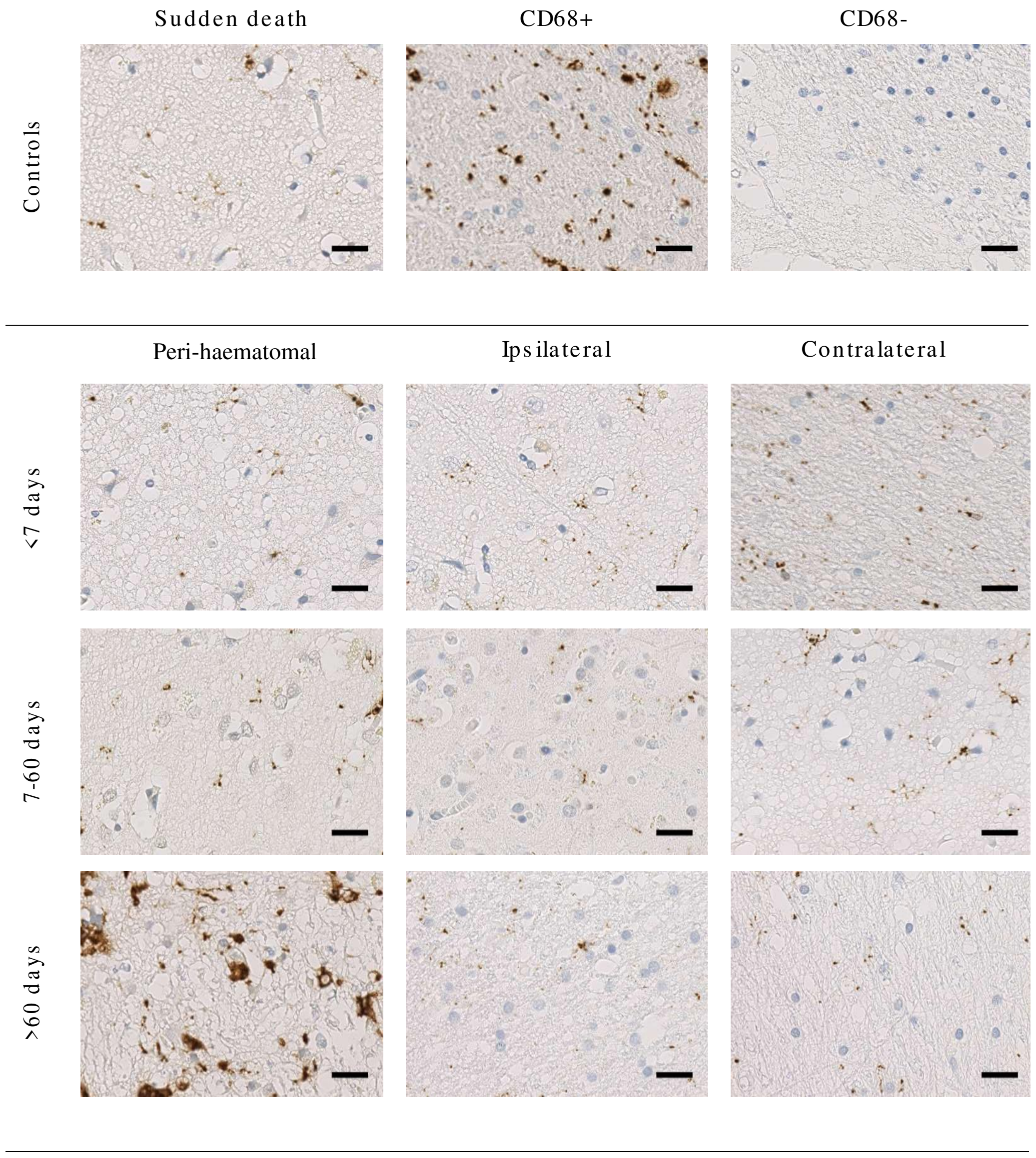

Supplementary figure 2. Representative images of CD68 immunohistochemical staining from ICH cases and sudden death controls included in the study. CD68+ and CD68– controls are shown on top row of middle and right column respectively as positive and negative controls. Images are all without manipulation and under the same magnification. Scale bars=20µm.

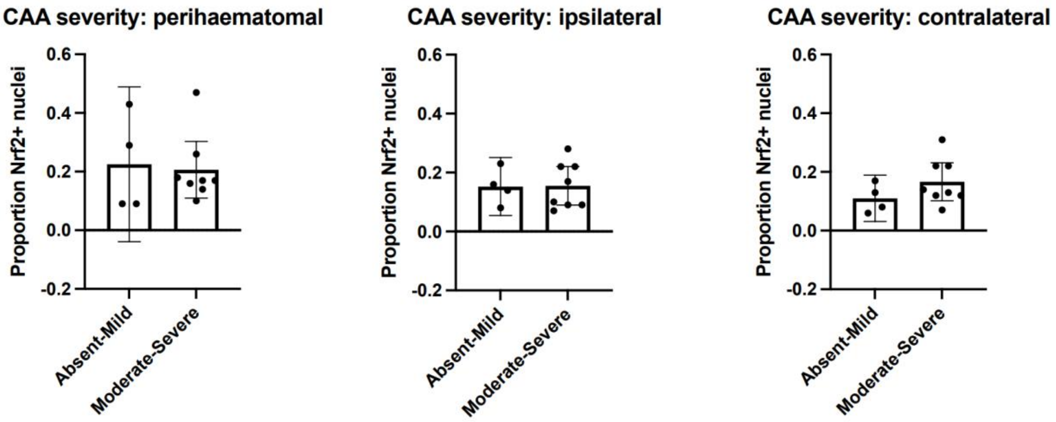

Supplementary figure 3. Mean (95% CI) proportion of nuclei stained positive for Nrf2 in lobar ICH tissue by location in relation to the ICH epicentre as well as cerebral amyloid angiopathy (CAA) ratings*. Our data do not suggest an association between CAA severity and Nrf2 nuclear localisation.

**Cerebral amyloid angiopathy rating was as previously defined in Rodrigues et al. The Edinburgh CT and genetic diagnostic criteria for lobar intracerebral haemorrhage associated with cerebral amyloid angiopathy: model development and diagnostic test accuracy study. The Lancet Neurology. March 2018; 17(3): 232-240.*
